# Baseline global brain structural and functional alterations at the time of symptom onset can predict subsequent cognitive deterioration in drug-naïve first-episode schizophrenia patients: Evidence from a follow-up study

**DOI:** 10.3389/fpsyt.2022.1012428

**Published:** 2022-10-14

**Authors:** Chuanjun Zhuo, Guangdong Chen, Jiayue Chen, Lei Yang, Qiuyu Zhang, Qianchen Li, Lina Wang, Xiaoyan Ma, Yun Sun, Feng Jia, Hongjun Tian, Deguo Jiang

**Affiliations:** ^1^Key Laboratory of Sensory Information Processing Abnormalities in Schizophrenia (SIPAS_Lab), Tianjin Fourth Center Hospital, Nankai University Affiliated Tianjin Fourth Center Hospital, Tianjin Medical University Affiliated of Tianjin Fourth Center Hospital, Tianjin, China; ^2^Department of Psychiatry, Wenzhou Seventh Peoples Hospital, Wenzhou, China; ^3^Department of Psychiatry, Tianjin Anding Hospital, Tianjin Mental Health Center of Tianjin Medical University, Nankai University Affiliated Tianjin Anding Hospital, Tianjin, China

**Keywords:** schizophrenia, ΔgGMV, ΔgFCD, cognition, correlation

## Abstract

Alterations in the global brain gray matter volume (gGMV) and global functional connectivity density (gFCD) play a pivotal role in the cognitive impairment and further deterioration in schizophrenia. This study aimed to assess the correlation between alterations in the gGMV and gFCD at baseline (ΔgGMV and ΔgFCD), and the subsequent alterations of cognitive function in schizophrenia patients after 2-year antipsychotic treatment. Global-brain magnetic resonance imaging scans were acquired from 877 drug-naïve, first-episode schizophrenia patients at baseline and after two years of antipsychotic treatment with adequate dosage and duration, and 200 healthy controls. According to ΔgGMV at baseline, schizophrenia patients were divided into mild, moderate, and severe alteration groups. The MATRICS consensus cognitive battery and Global Deficit Score (GDS) were used to assess cognitive impairment. We found that ΔgGMV and ΔgFCD at baseline were significantly correlated with the severity of the cognitive deterioration (ΔGDS). The correlation coefficient indicated a significant positive correlation between baseline ΔgFCD and subsequent cognitive deterioration, with a relatively stronger relation in the mild alteration group (*r* = 0.31). In addition, there was a significant positive correlation between baseline ΔgGMV and subsequent cognitive deterioration, with a stronger relation in the moderate and severe alteration groups (*r* = 0.303; *r* = 0.302, respectively). Our results showed that ΔgGMV and ΔgFCD are correlated with the severity of cognitive deterioration after completion of a 2-year antipsychotic treatment in schizophrenia patients. These findings suggest that baseline alterations in gGMV and gFCD hold potential for predicting subsequent cognitive decline in schizophrenia.

## Introduction

The neurodevelopment hypothesis of schizophrenia, suggesting that disruption of brain development in early life increases the risk of later developing of schizophrenia, was initially proposed in the early years of the 21st century. In the past 30 years, this hypothesis has matured sufficiently to incorporate childhood and adult adversity, urban living and migration, as well as heavy cannabis use, as important risk factors (1–3). Currently, the neurodevelopmental hypothesis of schizophrenia has morphed into the developmental risk factor model, after taking into account multiple lines of evidence that schizophrenia is not a discrete disease entity rather the severe end of a broader multidimensional psychosis spectrum (3–5). Numerous studies have demonstrated that there exists a continuum of subclinical psychotic symptoms, often associated with subtle cognitive deficits, extending into the general population and that the same factors that influence risk of schizophrenia also influence the prevalence of minor psychotic symptoms in the general population (6–9). Recent studies have provided further evidence in support that subtle cognitive and motor impairments appear early in life and that an abnormal neural maturation process increases the risk of developing schizophrenia spectrum disorders (10–14).

At present, cognitive impairment is viewed as a central feature of schizophrenia, affecting approximately 80% of patients, and impaired cognitive functioning represents one of the main obstacles to clinical and functional recovery (15–17). Interestingly, brain structural and functional alterations were observed in any stage of schizophrenia, and usually accompanied with cognitive impairments (18–26). Mounting evidence converges that cognitive impairment arises before the onset of psychotic-like symptoms and may play a pivotal role in the onset, development and prognosis of schizophrenia (10–14, 27). However, identifying cognitive impairments that precede the onset of illness is arduous, because cognitive deficits at the early stages of disease are subtle, but become increasingly pronounced as the patient’s progress from the prodromal phase to the first episode of psychotic symptoms (28–30). The rate at which patients show cognitive deterioration has been shown to differ among schizophrenia patients from the first episode of psychotic symptoms, experiencing mild to severe cognition impairment, especially over the first two years of antipsychotic treatment, even with adequate dosage and duration (27, 31–36).

The past three decades of research have witnessed substantial advances in magnetic resonance imaging (MRI) and functional magnetic resonance imaging (fMRI) techniques which allowed examining of the course of cognitive deterioration, enabling to identify of pre-onset brain abnormalities associated with the subsequent development of cognitive deficits. Therefore, it has been proposed that brain abnormalities preceding the onset of cognitive decline can be detectable with neuroimaging techniques; these biomarkers can be potentially used for early identification and prevention of progressive cognitive impairment in patients with schizophrenia (31, 37–40).

Cognitive impairment refers to deficits in the neurocognitive domains, including complex attention, executive function, learning, and memory, language, perceptual-motor, and social cognition (36, 41, 42). Based on neurodevelopment hypothesis of schizophrenia, cognitive impairment can be viewed as a reflection of the brain’s functional and structural abnormalities, especially within the brain circuits involved in information processing (43–45). Many neuroimaging studies support the notion that brain gray matter volume (GMV) abnormalities and brain functional connectivity density (FCD) disturbances in the whole brain can be the neural basis of cognitive impairments (18, 24, 25, 29–40, 46–54). However, until now, few studies have reported the relationship between the baseline global brain gray matter volume (gGMV) and global functional connectivity density (gFCD) and the subsequent cognitive deterioration in drug-naïve first-episode patients with schizophrenia following a two-year antipsychotic treatment with adequate dosages and adherence. Understanding the associations between the baseline gGMV and gFCD alterations and subsequent illness-associated cognitive deterioration in patients with schizophrenia can potentially provide clinically meaningful information that can optimize treatment strategies and slow down (or delay) further cognitive deterioration. Therefore, in the present study we explored the relationship between baseline neuroimaging parameters and subsequent illness-associated cognitive deterioration with the aim to identify possible prognostic biomarkers for patients with schizophrenia.

The global brain GMV alterations (ΔgGMV) represent the entire brain gray matter abnormalities, and ΔgGMV has been shown to be associated with cognitive impairments (46, 55, 56). The entire brain gFCD refers to the functional connection number in the entire brain, can reflect the information communicates capability of the entire brain, and play a pivotal role in the cognitive processing, the gFCD alterations (ΔgFCD) represent the entire brain function abnormalities (57–59).

In the present study, using ΔgGMV and ΔgFCD as indices, we aimed to determine the relationship between the baseline ΔgGMV and ΔgFCD values and the subsequently occurred cognitive deterioration following antipsychotic treatment with adequate dosage and duration in drug-naïve first-episode schizophrenia patients. We were interested in testing the following hypotheses: (1) Baseline ΔgGMV or ΔgFCD may be correlated with the subsequently occurred cognitive deterioration; (2) The relationship between ΔgGMV/ΔgFCD and deterioration of cognitive function as detected with changes of Global Deficit Score (ΔGDS) from baseline may have consistent correlation tendency in the patients with schizophrenia. The findings through conducting this study may provide a clue for predicting deterioration of cognitive function following initiation of antipsychotic treatment, and thereby assist psychiatrists in the future in finding optimal treatment strategies for cognitive deficits at early stages of the illness.

## Materials and methods

### Participants and procedures

Nankai University Affiliated Tianjin Fourth Center Hospital approved this study. A total of 1000 drug-naïve first episode patients with schizophrenia and 200 healthy controls were enrolled in this study. At baseline, qualitative MRI and fMRI data were acquired from 877 patients and 171 healthy controls. The social-demographical characteristics, illness information, and cognitive performance of the 877 patients and 171 healthy controls were presented in Table 1. After acquiring the MRI and fMRI data, all the patients received antipsychotic agents according to the Chinese Guidelines for the Prevention and Treatment of Schizophrenia. Upon completion of 2-year antipsychotic treatment with adequate dosage and duration, the patients were assessed for the secondary emerged cognitive impairments. A *post hoc* analysis was performed to examine the severity of cognitive deterioration.

**TABLE 1:** Social-demographical and clinical characteristics of all the study subjects.

	Schizophrenia patients (*n* = 877)	Healthy controls (*n* = 177)	*ANOVA/ACONAV* *P*
Age (mean ± SD), years	28.2 ± 3.5	27.5 ± 4.0	0.036
Gender (male/female)	625/252	115/62	0.028
Education level (years)	14.2 ± 5.0	16.8 ± 2.5	0.017
Illness duration (mean ± SD), days	107.2 ± 35.5		
PANSS (mean ± SD) at baseline	74.27 ± 5.45		
PANSS (mean ± SD) after two years treatment	53.24 ± 2.48		
MCCB (mean ± SD at baseline)	39.40 ± 2.77		
MCCB (mean ± SD) after two years treatment	32.85 ± 2.93		
GDS (mean ± SD) at baseline	0.95 ± 0.27		
GDS (mean ± SD) after two years treatment	0.68 ± 0.32		
gGMV (total altered voxel number, mean per person, which at baseline when compared to healthy controls)	19529.14 ± 1637.3		
gFCD (total altered voxel number, mean per person which at baseline when compared to healthy controls)	20071.29 ± 1300.83		

The symptom severity in schizophrenia was assessed by the Positive and Negative Syndrome Scale (PANSS), a well-established tool and widely used in the assessment of the illness severity and the efficacy of antipsychotic treatments for schizophrenia (60, 61). Cognitive impairments were evaluated by the MATRICS consensus cognitive battery (MCCB) (62), and the Global Deficit Score (GDS) was used for classification of overall impairment status on the MCCB battery plus a modified battery that included only those MCCB and added tests that were most sensitive to differences between patients with schizophrenia and healthy controls (35). MCCB was developed by The National Institute of Mental Health of the United States (NIMH) (63), and the tool evaluating seven cognitive domains that cover a wide range of neurocognitive functions is indeed a comprehensive reliable measurement to assess cognitive deficits in schizophrenia (64, 65). Several previous studies have used the MCCB to evaluate the levels of cognition in patients with schizophrenia due to its ideally psychometric properties (27, 66–68). In the past decades, many studies used MCCB to investigate the relationship between the cognitive performance and brain structural and functional alterations in the patients with schizophrenia (69–75). The Chinese version of the MCCB includes the following tests: Trail Making Test (TMT) Part A; Brief Assessment of Cognition in Schizophrenia (BACS) Symbol Coding; Form 1 of Hopkins Verbal Learning Test-Revised (HVLT-R), learning trials 1,2,3 and delay recall; Wechsler Memory Scale - Third Edition (WMS-III) Spatial Span; Form 1 of Neuropsychological Assessment Battery (NAB) Mazes; Form 1 of Brief Visuospatial Memory Test-Revised (BVMT-R), learning trials 1,2,3 and delay recall; Category fluency (animal names); Mayer-Salovey-Caruso Emotional Intelligence Test (MSCEIT) Managing Emotions; Continuous Performance Test-identical pairs version (CPT-IP). The MCCB coves seven cognitive domains: attention, information processing speed, verbal learning and memory, visual learning and memory, working memory, reasoning, problem solving, and social cognition (64, 66). GDS method can be described briefly as follows: demographically corrected T-scores were converted to deficit scores according to the following criteria: T > 39 = 0 (normal), 39 ≥ T ≥ 35 = 1 (mild impairment), 34 ≥ T ≥ 30 = 2 (mild to moderate impairment), 29 ≥ T ≥ 25 = 3 (moderate impairment), 24 ≥ T ≥ 20 = 4 (moderate to severe impairment), T < 20 = 5 (severe impairment). Deficit scores were summed across the test battery and then divided by the number of individual measures to compute the GDS. The GDS can be analyzed as a continuous variable indicating number and severity of neurobehavioral deficits across the entire test battery, or as a cut-off of ≥ 0.50 that can be used to classify overall neuropsychological impairment (76, 77). ΔGDS was defined as changes in GDS from baseline.

### Magnetic resonance imaging acquisition and analysis

An MRI scan was performed on a 3.0-Tesla MR system (Discovery MR750, General Electric, Milwaukee, WI, USA). T1-weighted images of all study participants were acquired with the following scanning parameters: repetition time (*TR*), 8.2 ms; echo time (*TE*), 3.2 ms; inversion time (*TI*), 450 ms; flip angle (*FA*), 12°; field of view (*FOV*), 256 × 256 mm; matrix, 256 × 256; slice thickness, 1 mm, no gap; and 188 sagittal slices (78). Resting-state (rs)-fMRI data were acquired using a gradient-echo single-short echo planar imaging sequence as reported previously, and the parameters were as follows: *TR*/*TE*, 2000/45 ms; *FOV*, 220 × 220 mm; matrix, 64 × 64; *FA*, 90°; slice thickness, 4 mm; gap, 0.5 mm; 32 interleaved transverse slices; and 180 volumes. During MRI scans, the patients and healthy controls received instructs to ensure safety and effective imaging as described previously (78).

### Gray matter volume calculation

T1-MPRAGE images were processed automatically using the Computational Anatomy Toolbox 12 (CAT12) extension of Statistical Parametric Mapping 12 (SPM12) running in MATLAB [2018b, Math Works, Natick, MA, United States, (79, 80)]. Image processing included bias field correction, skull dissection, alignment with the Montreal Neurological Institute standard space (MNI-152 template), and segmentation into GM, white matter (WM), and cerebrospinal fluid (CSF). A group-specific template was generated using the DARTEL algorithm (81). Segmented images in native space were then subjected to non-linear warping and normalized to match the DARTEL templates. Before preprocessing, all scans were visually inspected regarding artifacts and anatomical abnormalities by an experienced clinician. Structural MRI data were preprocessed using default parameters as implemented in the CAT12-Toolbox (Computation Anatomy Toolbox for SPM, build 1184. Structural Brain Mapping group, Jena University Hospital, Germany) building on SPM12 (Statistical Parametric Mapping, Institute of Neurology, London, UK), providing bias-corrected, tissue classified, and normalized data ratings. During preprocessing, images were segmented into GM, WM, and CSF. Images were spatially registered, segmented, and normalized using a DARTEL algorithm. All scans underwent the automated quality assurance, using the CAT12 “check data quality using covariance” procedure. After preprocessing and completing the quality assurance, we excluded 123 patients and 29 healthy controls due to major artifacts or anatomical abnormalities, or not fulfilling the CAT12 quality criteria, leaving 877 patients and 171 health controls for analysis in the current study.

### Functional magnetic resonance imaging image processing

Resting-state (rs)-fMRI scans were preprocessed using the Statistical Parametric Mapping (SPM) software, SPM12. To allow the signal to reach equilibrium and the study subjects to adapt to the scanning noise, the first 10 volumes for each participant were discarded. Subsequently, the volumes were corrected for the delay in acquisition time between slices, followed by realignment to correct the motion between time points. All rs-fMRI data were within the thresholds of the defined motion (i.e., translational or rotational motion parameters less than 2 mm or 2°). Frame-wise displacement (FD), which indexes the volume-to-volume changes in head position, was calculated. Several nuisance covariates were regressed out, including six motion parameters, first-time derivations, and average BOLD signals of the ventricular and white matter. Given the recent report that the signal spike due to head motion affected the final rs-fMRI data (82), we further regressed out spike volumes if the FD of the specific volume was greater than 0.5. The datasets were then band-pass filtered in a frequency range of 0.01–0.08 Hz. During normalization, the structural images were linearly co-registered with the mean functional image, and then linearly co-registered to MNI space. With co-registration parameters, each filtered functional volume was spatially normalized to MNI space and resampled into a 3-mm cubic voxel.

### Global functional connectivity density calculation

The data preprocessing for resting-state fMRI was performed by the Statistical Parametric Mapping (SPM12)1 in MATLAB 2014a (Mathworks, Inc., Natick, MA, United States). The first 10-time points were discarded, slice-timing correction, head motion estimation, normalization to standard Montreal Neurological Institute (MNI) EPI template and spatial smoothing with a 6-mm, and full-width-at-half-maximum Gaussian kernel were performed for the remaining 240-time points. Nuisance covariates regression was applied including six-direction head motion parameters, white matter, and cerebrospinal fluid (83). The full low frequency (FLF) of 0.01–0.08 Hz was performed for functional connectivity analysis. Based on the study of Rogachov et al. (84) and our previous study (85) on frequency-related neuroimaging studies of chronic pain, three different frequency band-based filters were selected for analysis, including Slow-5 band (0.01–0.027 HZ), Slow-4 band (0.027–0.073 Hz), and Slow-3 band (0.073–0.198 Hz).

### Global functional connectivity density calculation

The FCD was calculated by the BRANT toolkit in MATLAB 2014a. The FCD of each voxel was calculated according to the method described by Tomasi and Volkow (86–88). The gFCD value for a given voxel is the total number of active functional connections possessed by the voxel. Fisher *Z*-transformed version of correlation coefficient was the normalization method for FCD matrix. Pearson linear correlation analysis was performed to calculate the linear correlation between a given voxel (*i*) and all other voxels in the whole-brain as the number of global functional connections *k* (*i*), at a given voxel (*i*). Voxel pairs with a correlation coefficient of *r*_0_ > 0.6 were considered a significant connection. The gFCD calculations were limited to the cerebral gray matter mask _(*N voxels*)_ region, setting a signal-to-noise ratio greater than 50% to minimize the adverse effects of signal loss and artifacts associated with magnetic sensitivity (89).

Group analysis was applied using a random-effects model at different frequency bands. First, a voxel-based paired *t*-test was performed to measure the change in gFCD before and after the treatment in the VA or SA groups. Second, the brain regions that decreased or increased significantly after the treatment in the VA group compared with the SA group were explored by RMANOVA. Age was considered as a covariate in the statistics. For brain regions explicitly associated with pain in the previous studies that could not be corrected by family-wise error (FWE), a small-volume (anatomical structure) correction based 3dClustSim was taken by AFNI version 18.0.25 (90). The threshold of voxel-wise *p* < 0.005 and *p* < 0.05 FWE corrected at cluster level (more than 20 consecutive voxels) was applied for all the analyses.

### ΔgGMV and ΔgFCD calculation

The main objectives of the present study was to investigate the relationship between the secondary emerged cognitive deterioration and the baseline global brain GMV or functional features of the drug-naïve schizophrenia patients at the time of their first episode of psychosis. We calculated ΔgGMV and ΔgFCD in the whole brain at baseline using the following formula:

ΔgGMV = Increased GMV voxels (compared to the healthy controls at baseline) + decreased GMV voxels (compared to healthy controls at baseline). The severity of ΔgGMV was stratified by baseline ΔgGMV, compared to healthy controls, 0.5 ≤ ΔGMV < 1% defined as mild gGMV alterations, 1 ≤ ΔgGMV < 2% defined as moderated gGMV alterations, ΔgGMV ≥ 2% defined as severe gGMV alterations. Similarly, the following formulation was used to calculate ΔgFCD:

ΔgFCD = Increased gFCD voxels (compared to the healthy controls at baseline) + the decreased gFCD voxels (compared to healthy controls at baseline). Secondary emerged cognitive deterioration calculation In this study, the secondary emerged cognitive deterioration was calculated as follows:

Baseline MCCB scores transformed GDS score before treatment – MCCB score transformed GDS score after two years of treatment with adequate dosage of antipsychotic agents and duration.

The following formulation was used to calculate ΔPANSS:


ΔP⁢A⁢N⁢S⁢S=⁢P⁢A⁢N⁢S⁢S⁢a⁢t⁢b⁢a⁢s⁢e⁢l⁢i⁢n⁢e-P⁢A⁢N⁢S⁢S⁢a⁢f⁢t⁢e⁢r⁢t⁢w⁢o y⁢e⁢a⁢r⁢s⁢o⁢f⁢t⁢r⁢e⁢a⁢t⁢m⁢e⁢n⁢t.

ΔG⁢D⁢S=G⁢D⁢S⁢a⁢t⁢b⁢a⁢s⁢e⁢l⁢i⁢n⁢e-G⁢D⁢S⁢a⁢f⁢t⁢e⁢r⁢t⁢w⁢o⁢y⁢e⁢a⁢r⁢s⁢o⁢f⁢t⁢r⁢e⁢a⁢t⁢m⁢e⁢n⁢t.

### Statistical analysis

The statistical analysis was conducted using the statistical package SPSS Statistics version 25.0 (IBM Corp., Armonk, NY, USA). An analysis of variance (ANOVA) was used to determine the difference of means of the different groups: the mild ΔgGMV group (*n* = 259); the moderate ΔgGMV group (*n* = 385); the severe ΔgGMV group (*n* = 233), and the healthy control group (*n* = 117). The variables evaluated were age, gender, education level. PANSS, total cumulative dosage of antipsychotic agents within the two years of normalized antipsychotic treatment, cognitive impairment differences (ΔGDS), and baseline brain altered differences. Differences of ΔgGMV or ΔgFCD between groups were examined using one-way analysis of covariance (ANCOVA), where age, sex, and the mean FD were used as covariates. Regarding *post hoc* analyses, ANOVA were performed on significant group effects identified by ANCOVA. The family-wise error (FWE) was used to correct for multiple comparisons in both the ANCOVA and the *post hoc* analyses (*q* < 0.05) as described previously (91). Pearson correlation analysis was conducted to examine relationship between the baseline brain structural and functional alterations and the subsequent cognition impairment after two years of treatment with adequate antipsychotic agents in the schizophrenia patients.

## Results

### Baseline cognitive impairment subsequently deteriorated after antipsychotic treatment

All 877 schizophrenia patients showed a decrease in ΔGDS, indicating subsequent deterioration of cognitive function after two years of antipsychotic treatment with adequate dosage and duration. After 877 patients, 259 patients (259/877, 29.53%) had cognitive deterioration as detected with ΔGDS ≥ 0.5 (mean, 0.52; SD, 0.14); 385 patients (385/877, 44.92%) showed deterioration of cognitive function as examined with 1 ≤ ΔGDS > 0.5 (mean, 0.73; SD, 0.09); and the remaining 233 patients (233/877, 26.57%) had cognitive deterioration as detected with ΔGDS ≥ 1 (mean, 1.24; SD, 0.10). Despite 2-year antipsychotic treatment with adequate dosage and duration and effective amelioration of their psychotic symptoms (PANSS baseline – PANSS after two years of treatment) in nearly 63% of patients, there was a sharp decline in the cognitive function of these patients (Tables 1, 2).

**TABLE 2:** Clinical characteristics of patients in different groups and healthy controls.

	Patients with mild Δ gGMV (*n* = 259)	Patients with moderate Δ gGMV (*n* = 385)	Patients with severe Δ gGMV (*n* = 233)	*ANOVA/ACONAV* *P*
ΔPANSS (mean values of per person, which compared to baseline and two years)	24.27 ± 9.45	19.00 ± 9.59	14.87 ± 7.36	0.003
TCDA (mg, The accumulative dose of two years treatment, Chlorpromazine equivalent)	288457.52 ± 132122.43	375259.50 ± 1691542.91	596039.07 ± 299005.75	< 0.0001
ΔgGMV (mean voxel numbers of per person which compared between baseline and after two years treatment)	4529.14 ± 1637.52	6610.07 ± 1700.53	8887.62 ± 2003.44	< 0.0001
ΔgFCD (mean values of per person, which compared to baseline and two years treatment)	8256.72 ± 2459.04	52551.12 ± 2306.42	4007.25 ± 34300.85	< 0.0001
ΔMCCB (mean values of per person, which compared to baseline and two years treatment)	3.56 ± 0.98	5.28 ± 1.36	7.00 ± 0.98	0.007
ΔGDS (mean values of per person, which compared to baseline and two years)	0.52 ± 0.14	0.73 ± 0.09	1.24 ± 0.15	0.013

Data were presented as mean ± standard deviation (SD). TCDA, total cumulative dosage of antipsychotic agents within the 2 year normalized treatment, Chlorpromazine equivalent.

### Subsequent cognitive deterioration after completion of treatment was not correlated with the therapeutic effects on psychotic symptoms

We performed a Spearman correlation analysis, and unexpectedly the resulting data did not demonstrate any significant correlation between therapeutic effects on psychotic symptoms/the cumulative dosage of antipsychotic agents within the two years of normalized treatment and the subsequent cognitive deterioration in the drug-naïve first-episode schizophrenia patients. Subsequent cognitive deterioration after completion of treatment was positively.

### Correlated with baselineΔgGMV/ΔgFCD

**FIGURE 1:**
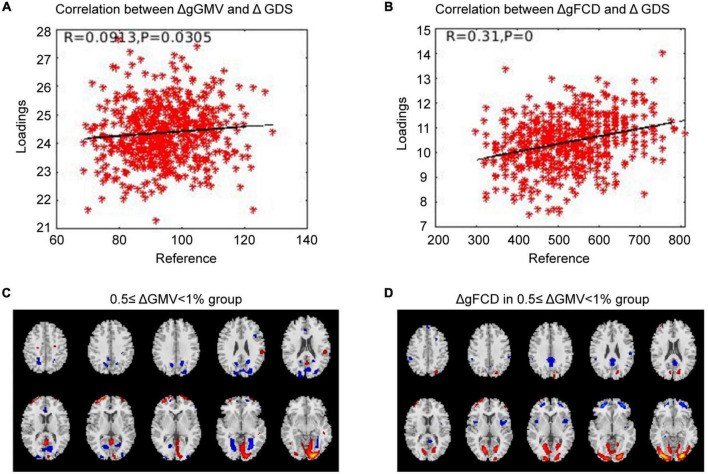
The correlation between ΔGMV/ΔgFCD and the cognitive deterioration in the mild whole-brain alteration group. Correlation analysis was performed to determine the relationship between subsequent cognitive decline after two years of treatment and baseline ΔgGMV/ΔgFCD in the drug-naïve first-episode schizophrenia with 0.5 ≤ ΔgGMV < 1% at baseline in the mild whole-brain alteration group. **(A)** Relationship between ΔgGMV and ΔGDS; **(B)** Relationship between ΔgFCD and ΔGDS; **(C)** The mild whole-brain alteration group with 0.5 ≤ ΔgGMV < 1% at baseline; **(D)** ΔgFCD in the mild whole-brain alteration group.

**FIGURE 2:**
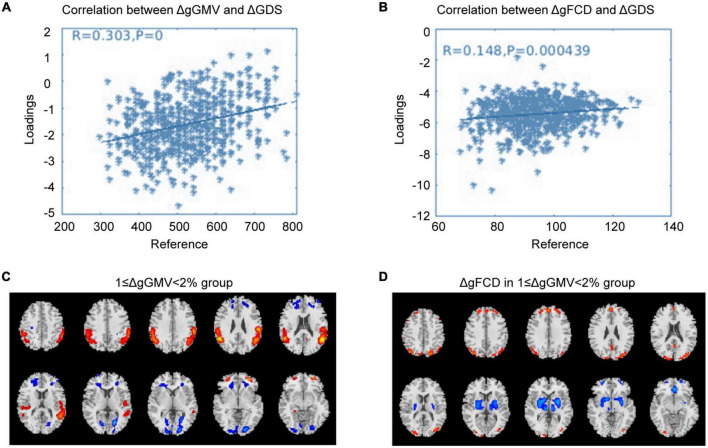
The correlation between ΔGMV/ΔgFCD and cognitive deterioration in the moderate whole-brain alteration group. Correlation analysis was carried out to evaluate the relationship between subsequent cognitive decline after two years of antipsychotic treatment and baseline ΔgGMV/ΔgFCD in the drug-naïve first-episode schizophrenia with 1 ≥ ΔwGMV < 2% at baseline in the moderate whole-brain alteration group. **(A)** Relationship between ΔgGMV and ΔGDS; **(B)** Relationship between ΔgFCD and ΔGDS; **(C)** The moderate whole-brain alteration group with 1 ≥ ΔwGMV < 2% at baseline; **(D)** ΔgFCD in the moderate whole-brain alteration group.

**FIGURE 3:**
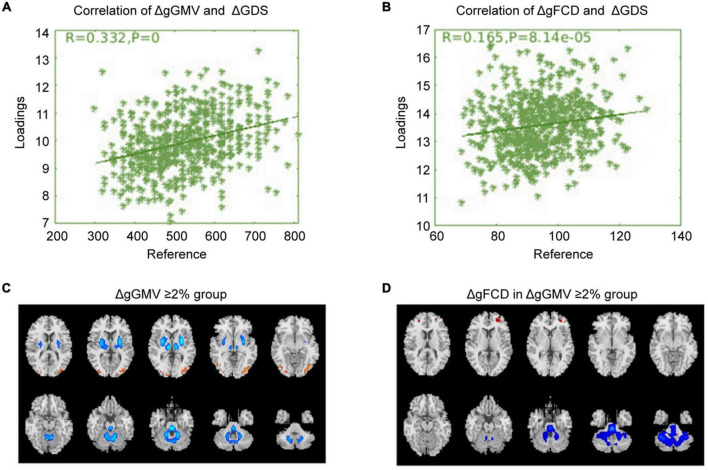
The correlation between baseline ΔgGMV/ΔgFCD and cognitive deterioration in the severe whole-brain alteration group. Based on ΔgGMV at baseline, schizophrenia patients were divided into three different severity groups: mild, moderate, and severe brain alteration groups. Correlation analysis was conducted to examine the relationship between subsequent cognitive deterioration after two years of treatment and baselineΔgGMV/ΔgFCD in the drug-naïve first-episode schizophrenia with baseline ΔgGMV ≥ 2% in the severe whole-brain alteration group. **(A)** Relationship between ΔgGMV and ΔGDS; **(B)** Relationship between ΔgFCD and ΔGDS; **(C)** The severe whole-brain alteration group with ΔgGMV ≥ 2% at baseline; **(D)** ΔgFCD in the severe whole-brain alteration group.

Correlation analysis was carried out to examine the relationship between subsequent cognitive deterioration after completion of treatment and altered ΔgGMV/ΔgFCD at baseline in the different severity of brain alteration groups. As shown in Figures 1–3, the correlation coefficient indicated a positive correlation between baseline ΔgGMV/ΔgFCD and subsequent cognitive deterioration. More interestingly, in this group, ΔgFCD demonstrated a relatively stronger relation than ΔgGMV was found in schizophrenia patients with mild brain alterations [Figure 1, *r* = 0.31(ΔgFCD) vs. *r* = 0.091(ΔgGMV)]. Furthermore, there was a positive correlation between baseline ΔgGMV and subsequent cognitive deterioration, and a stronger relation was observed in schizophrenia patients with moderate [Figure 2, *r* = 0.148(ΔgFCD) *vs*. *r* = 0.303(ΔgGMV)] and severe brain alterations [Figure 3, *r* = 0.165(ΔgFCD) vs. *r* = 0.336(ΔgGMV)]. Collectively, the data revealed different degrees of correlation between baseline ΔgGMV and subsequently deterioration of cognitive function after antipsychotic treatment with adequate dosage and duration among drug-naïve first episode schizophrenia patients.

## Discussion

The present study with a relatively large sample size has the following major novel findings that may lead future studies to clues for predicting and understanding subsequent cognitive deterioration after antipsychotic treatment in drug-naïve first-episode schizophrenia patients: (1) The cognitive function of the patients with schizophrenia demonstrated a tendency of subsequent deterioration from psychotic symptom onset to completion of two years of antipsychotic treatment despite adequate antipsychotic medication dosages and treatment duration; (2) Baseline altered values of global brain GMV (ΔgGMV) were correlated with the subsequent deterioration of cognitive function after two years of antipsychotic treatment with adequate dosage and duration; (3) Baseline whole-brain functional alterations (ΔgFCD) were also correlated with the subsequently occurred cognitive deterioration after two years of antipsychotic treatment with adequate dosage and duration.

Previous studies reported that some of antipsychotic agents, especially second generation antipsychotics, can improve the cognitive function of the patients with schizophrenia (92–96). Unfortunately, the schizophrenia patients in the present study who received treatment with the second generation antipsychotic agents according to the schizophrenia treatment guideline did not demonstrated improvement of cognitive function. However, some previous neurocognitive studies reported that cognitive impairment, once occurred, cannot be reversed by drugs, even after treatment with neuroprotective agents, and all the drugs which aimed at improving the cognitive deficits only had minimal effects delaying the deterioration of cognitive decline (97–101). Our data have provided evidence in support of this opinion. At the same time, a number of previous studies showed that the antipsychotic agents cannot reverse the cognitive impairment (102–105). Our data did not support the earlier findings that antipsychotic agents can reverse the cognitive deficit in patients with schizophrenia, as it was reported in the randomized controlled trial (RCT) studies (97–101). Additional studies are needed to gain new evidence for the protective effect of antipsychotic agents on the cognitive deficit.

It may merit attention in this study that baseline altered whole-brain GMV and functional alterations (ΔgGMV/ΔgFCD) were correlated with the subsequent cognitive deterioration. To the best of our knowledge, the interesting finding has not been reported, and baseline ΔgGMV/ΔgFCD may provide a clue for predicting decline of cognitive function even after antipsychotic treatment. Despite the strength, we think some questions need to be answered. First, the whole-brain GM structural alterations represented the sum of increased GMV and decreased GMV. Previous studies reported that decreased GMV was usually associated with the decreased cognitive deficit and poor therapeutic effect. In contrast, increased GMV usually represents the alleviating of cognitive deficit and is usually associated with a better therapeutic effect. To date, few studies reported on the use of increased GMV and decreased GMV in combination as a general alteration of the whole brain to investigate the relationship between the whole-brain GMV changes and the cognitive alterations. Similarly, increased gFCD usually represents higher information communication, while decreased gFCD usually reflects lower information communication in the brain. Few studies have been conducted to assess the relationship between the whole-brain gFCD changes and cognitive alterations. Previous studies found that the whole brain was involved in processing the complex information (18, 20, 106–108). As aforementioned, cognitive function involves important information processing, including complex attention, executive function, learning, and memory, language, perceptual-motor, and social cognition. It has been considered that the full cognitive function is a complex phenomenon that needs many brain regions to coordinate activities, including functionally activated and suppressed specific brain regions to ensure understanding and handling information with precision (18, 20, 106–108). According to the whole brain functional activity involved in the cognitive processing theory, the whole-brain structural and functional alterations are anticipated to influence cognitive information processing, and in turn cause cognitive disturbance. In light of this view, the correlation between baseline ΔgGMV and ΔgFCD and subsequent cognitive deterioration in this study may have neural and theoretical basis.

It was also worth noting in this study that the correlation degree differed among three groups with different baseline severity of brain alterations as classified by baseline MRI/fMRI features. In the severe brain GMV alteration group, the correlation coefficient between ΔgGMV and ΔGDS was 0.332, and that between ΔgFCD and ΔGDS was 0.165, suggesting that the cognitive deterioration was mainly correlated to the baseline whole-brain GMV alterations. In the moderate brain alteration group, the co-efficient between ΔGMV and ΔGDS was 0.303, and that between ΔgFCD and ΔGDS was 0.148. The results from correlative analysis in both severe and moderate brain alteration groups indicated that the whole-brain GMV alterations at baseline seemed to play the crucial role in the subsequent cognitive deterioration. However, in the mild brain alteration group, the co-efficient between ΔGMV and ΔGDS was 0.093, and that between ΔgFCD and ΔGDS was 0.310, suggesting that the whole-brain functional connection numbers play a role in the cognitive deterioration in schizophrenia patients with mild brain alterations at baseline. With these data, we were inclined to think that the whole-brain GM alteration (ΔgGMV) may be the neural basis of the cognitive deterioration, and the greater value of ΔgGMV may predict a higher degree of cognitive deterioration. In the mild brain alteration group, the co-efficient between ΔgGMV and cognitive decline was lower than that of the ΔgFCD and the cognitive deterioration, for which we postulated that the ΔgFCD might be the functional compensation (109, 110) to the ΔgGMV. Thus the correlation co-efficient of ΔgFCD and ΔGDS was higher than that of ΔGMV and ΔGDS. However, when in the moderate and severe ΔGMV groups, the functional compensation of ΔgFCD cannot enough to strength to make up the cognitive deficit caused by ΔgGMV. With our findings and those of others, we are inclined to think that antipsychotic agents do little to improve the cognition impairment in patients with schizophrenia, and even worse than that the medications have been shown to be associated with worsening in cognitive capacity (100, 111). Notably, mounting studies reported that psychosocial treatments acquired the cognitive remediation effect in the patients with schizophrenia. For example, there are several approaches to cognitive remediation. Core features include using cognitive training techniques, typically computerized to enhance neuroplasticity; therapist-guided development and refinement of problem-solving strategies that can be used during cognitive training and in daily life; and facilitating the transfer of cognitive gains and new strategies to daily life (93, 112, 113). However, the cognition remediation is not the same as cognition recovery and has limitations. Up to date, few studies have shown that cognitive remediation can help recover cognition to the pre-onset level in patients with schizophrenia. As proposed by the Cognitive Remediation Expert Working Group (CREW) “Cognitive remediation is now widely recognized as an effective treatment for cognitive deficits in schizophrenia. Its effects are meaningful, durable, and related to improvements in everyday functional outcomes (112).” Collectively, cognition impairment is worsening progressively in schizophrenia that has posed therapeutic challenges, and new effective treatments are needed to improve medical care for patients with schizophrenia.

We realized a number of limitations in this study. First, the use of ΔgGMV and ΔgFCD as brain structural and functional alterations in this study has not been reported and will need validation. Second, ΔgGMV and ΔgFCD in the present study was calculated as the sum of altered gGMV and gFCD in the patients compared to the healthy controls at baseline. Similarly, the validity of the calculation will be needed in future studies. Third, the threshold values of ΔgGMV for severity classification of three different brain alteration groups had no references to use, hence the validity of this method is also needed in a future study. Forth, we could not explain some observations in this study, including that there was no relationship between the cumulative dosage of antipsychotic agents and cognitive function deterioration. Hence, further in-depth investigations will be needed in future studies.

## Conclusion

Taken together, this is the first report on the relationship between the baseline brain alterations and the subsequently occurred cognitive deterioration in drug-naïve first-episode schizophrenia patients. Our findings have demonstrated that the altered whole-brain GMV at baseline significantly correlates with the subsequent decline of cognitive function, especially moderated to severe cognitive deterioration. Unexpectedly, the findings have revealed no correlation between cognitive function deterioration, therapeutic effects, and the cumulated dosage of anti-psychotic agents during the 2-year normalized treatment. As such, this study has provided a clue for a better understanding of the deterioration of cognitive function in patients with schizophrenia. In addition, the new findings may have the clinical implication that baseline altered gGMV and gFCD values hold the potential to predict subsequent cognitive deterioration in drug-naïve first-episode schizophrenia patients.

## Data availability statement

The original contributions presented in this study have been included in the article. Further inquiries can be directed to the corresponding authors.

## Ethics statement

The studies involving human participants were reviewed and approved by Tianjin Fourth Center Hospital. The patients/participants provided their written informed consent to participate in this study.

## Author contributions

CZ, GC, and JC: conceptualization, methodology, software, analysis, investigation, and writing – original draft preparation. LY, QZ, QL, and LW: software, analysis, and writing – review and editing. XM, YS, and FJ: software, investigation, and writing – review and editing. HT and DJ: conceptualization and supervision. All authors contributed to the article and approved the submitted version.
